# Risk perceptions of STIs/HIV and sexual risk behaviours among sexually experienced adolescents in the Northern part of Lao PDR

**DOI:** 10.1186/1471-2458-13-1126

**Published:** 2013-12-05

**Authors:** Vanphanom Sychareun, Sarah Thomsen, Kongmany Chaleunvong, Elisabeth Faxelid

**Affiliations:** 1 Faculty of Postgraduate Studies, University of Health Sciences, Samsenthia Road, P.O.Box 7444, Vientiane, Lao PDR; 2 Division of Global Health (IHCAR), Department of Public Health Sciences, Karolinska Institutet, Stockholm, SE 171 77, Sweden; 3 Faculty of Basic Sciences, University of Health Sciences, Samsenthia Road, P.O.Box 7444, Vientiane, Lao PDR

**Keywords:** Risk perceptions, Sexual risk behaviors, Luangnamtha province, Lao PDR

## Abstract

**Background:**

Young people in Laos are more vulnerable to STIs/HIV due to their sexual risk behaviours, low perceptions of risk and their socio-cultural environments. Perceived risk of contracting STIs/HIV is crucial for the assessment of their risk regarding their actual sexual risk behaviors. Thus, the objective of this paper is to explore perceptions of risk related to STIs/HIV and identify factors associated with this perceived risk among adolescents.

**Methods:**

This was a cross sectional study of sexually experienced adolescents aged 14 to 19 years old in the Luangnamtha province. The multistage sampling techniques were used for selecting 1008 adolescents aged 14-19 years old. Of these, 483 respondents reported having had sexual experience was selected for analysis. Univariate and Logistic regression were performed.

**Result:**

Six per cent of respondents reported ever having had anal sex. Slightly less than two thirds initiated their first sexual intercourse before age 15. Two thirds of the sexually experienced males reported two or more sexual partners during their lifetime with the mean 3.1 + 3.65 while only twelve per cent of girls reported this cumulative number of partners. Slightly more than half (57.6%) regarded themselves to have no risk at all with 17.2 per cent considered themselves to have low risk. Respondents had poor knowledge on STIs/HIV. Factors associated with risk perception of getting STIs were: being male, high level of knowledge about STIs and having had symptoms of STIs in last six months. Perceived risk of getting HIV was significantly associated with being male, having more knowledge about STIs and HIV.

**Conclusion:**

Adolescents in this study engaged in sexual risk behaviours, but they have low perception of risk getting STI/HIV. Socio-demographic factors, knowledge of STIs/HIV, and the level of exposure to STIs were the main determinants of the risk perception of STIs/HIV. Our finding supports the need to target adolescents in Luangnamtha province for HIV prevention intervention by addressing inaccurate perception of risk and increasing their knowledge on STIs/HIV.

## Background

HIV/AIDS is emerging as a potentially serious health issue for Lao PDR, even though, it is currently categorized as a low HIV prevalence country, with an estimated HIV prevalence of 0.2% [1]. At the mid of 2011, the official cumulative number of HIV positive people was 4,272. Of those, 2,736 were living with HIV/AIDS and 1,170 had already died [2]. However, Lao PDR has a high prevalence of sexually transmitted infections (STIs) (1.8%), especially among most-at-risk population [1]. STIs are the most pervasive infectious diseases confronting adolescents. Strong evidence supports several biological mechanisms through which STIs facilitate HIV transmission by increasing both HIV infectiousness and HIV susceptibility [3]. Thus if the incidence/prevalence of STIs is high in a country, there is a high risk of sexual transmission of HIV [4]. The major mode of STIs including HIV transmission in Lao PDR is heterosexual and the majority of people infected are men aged 20-29 years old [1]. The serious and growing epidemics in several of Lao PDR’s neighboring countries, increasing population mobility, both within and across Lao PDR’s borders, trafficking of women, girls and boys and improved transportation networks make the country increasingly vulnerable to HIV/AIDS [5].

The Lao National HIV/AIDS strategies 2011-2015 recognize that young people are a particular vulnerable group for HIV infection [6]. Adolescents aged 13 to 19 have been identified as a vulnerable group for acquiring STIs and HIV/AIDS, especially adolescents from ethnic minorities [7-9]. The vulnerability of adolescents to the HIV infection is due to personal [10-12] and socio-cultural factors [9,13]. Personal factors include lack of HIV knowledge and widespread misconceptions; limited knowledge of preventive methods, and greater biological susceptibility for girls to STIs/HIV infections and its consequences [14,15]. Sexual risk behaviours include initiation of sexual intercourse at an early age, multiple sex partners within a short period, and practice of unsafe sex such as no condom use [16,17] and presence of STI symptoms [18]. Sexual behaviour is dependent upon the social and cultural environment in which one lives, and is influenced by societal sexual norms and practices, and not just self-perceived susceptibility to HIV infection [19]. Socio-cultural factor includes traditional sexual customs such as “Vagina Breakthrough” and “Welcome Guest” for girls and “Open foreskin” for boys, which make them more at risk of STIs including HIV [9]. “Vagina Breakthrough” is a pre-pubertal rite of passage whereby a young girl has penetrative sexual intercourse with an older, sexually mature man. The act of “Vagina Breakthrough” makes a “hole” in the girl’s vagina making her sexually mature. Girls who have performed “Breakthrough Vagina” may then participate in the cultural practice of “Welcome Guest” whereby they entertain male visitors to the village by providing massage to the visitors in a small “Welcome Guest” hut. Boys aged 12 and 15 must seek out an older, sexually experienced woman for their first sexual encounter in order to “open” the foreskin and mature into a man.

The Health Belief Model (HBM) identifies the perceived susceptibility or perceived risk, perceived severity, perceived benefits, and perceived barriers as predictor of safer sexual activity [20]. Previous researchers have suggested that there is an association between higher level of sexual risk taking and high level perceived risks of contracting HIV [19,21-23]; however, some researchers have not found any association [24]. Other studies have found that low level of sexual risk-taking results from high levels of perceived risks of contracting HIV [25].

In addition to the HBM, other models of behavioural change have incorporated additional key constructs such as concept of self-efficacy, which is defined as having confidence in one’s ability to perform a particular behavior [26-28]. Self-efficacy plays a crucial role in STI/HIV prevention behavior such as using condoms and limiting the number of sexual partners. Positive perception of self-efficacy would be expected to be inversely associated with STIs/HIV risk behaviors.

Perceived peer sexual norms are significant determinant of the spread of STI/HIV/AIDS through the impact of permissive sexual norms on sexual behaviours [29,30]. Peer sexual norms play an important part role in sexual behaviour. Involvement in risky sexual practices was found to be higher among youth who have peers who hold permissive sexual norms [31]. Although social influences, particularly peer norms, seem to be important determinants of sexual initiation, the complex relationship between the different components of peer norms and behavioral change has yet to be clearly elucidated [32,33].

Relatively few studies in Lao PDR have examined adolescent’s sexual risk taking behaviors and its association with perceived risk of getting STIs/HIV. Thus, the objective of this paper is to explore perceptions of risk related to STIs/HIV and identify factors associated with this perceived risk among adolescents. Perceived risk of contracting STIs/HIV may have important implications for prevention programs among adolescents from different ethnic groups. This study is significant because it provides insights into the subjective perceptions of risk of STIs/HIV infection in a population identified as a vulnerable to HIV but with whom there has been limited research and situates risk perception within the socio-cultural context of northern Lao PDR. This focus is particularly pertinent given that the sexual behaviors of adolescents from ethnic minorities can expose them to the risks of STIs/HIV.

## Methods

### Study setting

The study was carried out in the rural areas of Luangnamtha province in Northern Lao PDR using a community-based sample of adolescents. Luangnamtha province has 32 ethnic groups, occupies an area of 9,325 square kilometers, and has a total population of 145,231 people with 73,873 females. Of the total population, 36,531 belong to the Akha ethnic group [34]. The population structure consists of predominantly young people, with the majority aged 15-30 years [35]. The net enrollment in the lower secondary school in the province is low compared to the national data (15% versus 28.5%) [36].

Long and Sing districts were the target study sites. These districts were selected because there are many different ethnic groups with about half belonging to the Akha ethnic group. There is also high poverty in these districts [37]. In addition, these districts share border with neighboring countries with high STI/HIV prevalence such as Myanmar and China (Yunnan province). Many ethnic groups still have some traditional sexual customs that put adolescents at significant risks related to sexual and reproductive health. These cultural beliefs might moderate the way in which STI/HIV is perceived and therefore addressed in that particular context [38].

The first case of HIV in Luangnamtha province was detected in 2004 and the province has emerged as the province with the highest prevalence of reported HIV infection (0.03%) in the country. According to the annual report of Luangnamtha Provincial Health Department in 2011, 2769 people have been tested for HIV. Forty-six were HIV positive, 30 lived with HIV/AIDS and 12 deaths had been recorded in Luangnamtha province by the end of 2010 [34]. In addition, the province has a high prevalence of STIs (4.3%) [1].

### Design and participants

This was a quantitative cross-sectional descriptive study. The estimated sample size yielded 400 males and 650 females based on the standard formula for the population proportion [39]. Two-stage systematic random sampling was employed to recruit the target group into the study. The sampling frame of adolescents aged 14-19 years in the selected villages was prepared from the household enumeration. While the World Health Organization defines adolescents as young people between the ages of 10 and 19 years [40], for the purpose of this study we used the age range of 14-19 as most adolescents in the target population begin sexual relations between 12-15 years [9,41]. First, the sampling list of villages in the two districts was prepared, then 40 villages were randomly selected with probability proportional to size of each district. Secondly, from each selected village, unmarried adolescents aged 14-19 years were systematically randomly selected from the constructed sampling frame with probability proportional to size of the villages. A total of 1050 adolescents were approached and 1008 were enrolled into the study, thus four per cent did not participate. The primary reasons for non-participation were being absent at the time of interview. Respondents were asked the following questions: a) Have you ever had sexual intercourse? b) Have you ever had vaginal intercourse? c) “Have you ever had anal intercourse? Vaginal sex was defined as penile vagina penetrative sexual intercourse; oral sex referred to involving penetration of the mouth by the penis or oral penetration of the vulva or vagina; and anal sex was defined as penetration of the anus by the penis. Then, they were classified as having sexual experience if they answered “yes” to one of the above questions. Among 1008 respondents, 486 were sexually experienced; however, 3 questionnaires were incomplete, thus, the sample for this analysis was restricted to the 483 adolescents who reported having had sexual experience.

### Measures

Respondents were asked about socio-demographic factors, psychosocial factors (self-efficacy and peer sexual norms), knowledge about STIs, including HIV, and markers of risky sexual behaviors (early age at first sexual intercourse, number of sex partners, unprotected sexual intercourse, and alcohol and drug use) (Additional file 1). Background characteristics of respondents included sex, age, education, currently going to school, ethnicity, and living arrangement. Ethnicity was measured by respondent’s identification as Lao, Khamu, Akha, Hmong, or Yao. Then, this variable was recorded as Akha and Non Akha. Socio-cultural practices such as experience of ‘Vagina Breakthrough’, ‘Welcome guest’ for girls and “Open foreskin” for boys were also asked. Risk factors for example, knowledge of STIs and HIV, practices such as condom use during the last 6 months and having STIs symptoms were identified as being important in the literature [14-18].

The section regarding STIs knowledge included whether the respondents had heard of STIs, major source of STIs information, STIs symptoms, transmission route, and consequences of untreated STIs. The correct response was coded 1 and each incorrect or non-response was coded 0. All 12 STIs questions were summed up with higher score indicating higher level of knowledge of STIs. The internal consistency of the scale Kuder-Richardson-20 (KR-20) in this sample was 0.67.

The section related to awareness of HIV included questions whether the respondent had heard of HIVAIDS and the major source of HIV information. We used the brief HIV knowledge Questionnaire (HIVKQ) [42,43] to assess knowledge regarding transmission and prevention of HIV which was modified according to the Lao context. Respondents answered to 8 items using true/false. Responses were scored 1 if correct and 0 if not. In total, therefore, scores on the HIV knowledge range from 0 to 8. All 8 HIV questions were summed up with higher score indicating higher level of knowledge of HIV. The internal consistency of the scale was KR-20 coefficient = .65.

Self-efficacy was measured using a scale score on abstinence, condom use, and communication with partner. This scale was constructed from a previously validated scale of self-efficacy [22,44] and included 15 items. Respondents rated each item using a 5 point scale ranging from not at all to very likely, with increasing scores indicating higher self-efficacy. The internal consistency of the scores on the 5-item scale, as measured using Cronbach’s alpha coefficient was .87.

The perceived peer sexual norms questionnaire, a self-reported measure, had five items originally developed in English by previous researchers [30,45]. The scale assessed what adolescents believed their friends thought about engaging in sexual risk behaviors. The adolescents were asked to indicate the degree to which an item described what they believed on a five-point Likert scale ranging from 0 (none of my friends) to 5 (all of my friends) engaged in sexual risk behaviors (having multiple sexual partners, no condom use, selling sex, having STIs, and using illicit drugs). This procedure is consistent with established methodologies for assessing perceptions of friends’ sexual behaviour [46]. The total score ranged from 0 to 14, with a Cronbach’s alpha coefficient of .79. Higher score indicated perception of more permissive sexual peer norms.

To determine the level of risk perception, participants were asked how afraid they were of getting STIs and HIV and the chance of contracting STIs and HIV. The response was categorized as 0 = “no chance” or 1 = “small chance” of contracting the disease and were classified as having low risk perception. Those reporting their risk as 3 = “somewhat” chance were classified as having moderate risk perception and those reporting or 4 = “high” chance or 5-“very high chance” were coded as “high risk”.

Sexual behaviors were obtained from the sexually experienced adolescents who reported ever having had sex. Questions included age at first sex, numbers and type of sexual partners to date, sexual experiences during the last six months prior to the interview, condom use at the first sex and during the last six months preceding the interview. Finally the respondents reported experience of having STI symptoms during the last six and 12 months.

#### Data collection

Experienced interviewers from the Faculty of Postgraduate Studies and Research underwent three days training about the objectives of the research project, data collection process, sampling procedures, and the content of the questionnaire. The questionnaire was translated from English into the local language Lao. In addition, young Akha translators were recruited to support data collection where Akha respondents were not comfortable in speaking Lao. Interviewers were matched with the sex of respondents and privacy was to ensure during interview.

### Data analysis

Data entry was done using EPI-INFO. The data were analyzed by using SPSS version 10.0 and STATA. Univariate analysis was performed by presenting frequencies and percentages for categorical variables and means (±Standard Deviation) for continuous variables. Results from male and female adolescents were compared. Unadjusted and adjusted OR with 95% confidence intervals (95% CI) was calculated in bivariate and multivariate analyses. Statistically significant variables in the bivariate analyses were entered into the multivariate model. Multivariate ordinal logistic regression models were employed to examine the factors associated with perceived risk of STIs and HIV, while controlling for some of the socio-demographic factors.

The research protocol was reviewed and approved by the Ethical Review Board at the University of Health Sciences, Vientiane, Lao PDR. Participants were informed about confidentiality and verbal consent was obtained. Parental consent was also obtained for those less than 18 years of age.

## Results

### Socio-demographic characteristics

The characteristics of the sexually experienced respondents (245 boys and 238 girls) are displayed in Table 1. The mean age of respondents was 16.4 + 1.65 years with a range of 14 to 19 years. Overwhelmingly the majority of respondents were from the Akha ethnic group (84.5%) and only 15.5 per cent were non-Akha. Results on the educational status showed 22.2 per cent were illiterate, and 48.2 per cent had finished primary school. Females reported lower education levels than males (37.8% versus 6.9%, p < .001). More than one third (39.3%) of adolescents were currently attending school with more boys than girls currently in school (51.8% versus 25.5%, p < .001). Almost all participants were living with parents (97.3%).

**Table 1:** Socio demographic characteristic of sexually experienced respondents, by selected characteristics, (n = 483)

	**Male (n = 245)**		**Female (n = 238)**		**Total (n = 483)**		**P value**
	**N**	**%**	**N**	**%**	**N**	**%**	
Age							< 0.001
14–15	73	29.8	109	45.8	182	37.7	
16–19	172	70.2	129	54.2	301	62.3	
Mean (Sd)	16.8 (1.64)		15.9 (1.55)		16.4 (1.65)		
Ethnicity							< 0.001*
Lao	52	21.2	4	1.7	56	11.6	
Khamu	1	0.4	0	0.0	1	0.2	
Hmong & Yao	17	6.9	1	0.4	18	3.7	
Akha	175	71.4	233	97.9	408	84.5	
Level of schooling complete							< 0.001*
Never go to school	17	6.9	90	37.8	107	22.2	
Primary School	119	48.6	114	47.9	233	48.2	
Secondary school	81	33.1	28	11.8	109	22.6	
Upper secondary school	27	11.0	4	1.7	31	6.4	
Technical	0	0.0	1	0.4	1	0.2	
University	1	0.4	0	0	1	0.2	
Current going to school							< 0.001
No	118	48.2	175	73.5	293	60.7	
Yes	127	51.8	63	26.5	190	39.3	
Living arrangement							0.124*
Family	235	95.9	235	95.9	470	97.3	
Dormitory	8	3.3	3	1.3	11	2.3	
Other	2	0.8	0	0.0	2	0.4	

**Note:** * = Fisher’s exact.

### Sexual risk behaviors

Six per cent of respondents reported ever having had anal sex (Table 2). Slightly less than two thirds initiated their first sexual intercourse before age 15. Two thirds of the sexually experienced males reported two or more sexual partners during their lifetime with the mean 3.1 + 3.65 while only twelve per cent of girls reported this cumulative number of partners. Forty two per cent reported not having used any contraceptives at all, 30.2 per cent reported using a condom, 20 per cent used other methods such as oral contraceptives or withdrawal method and 8 per cent reported that they used a combination of condoms and other methods.

**Table 2:** Sexual risk behaviors (n=483)

**Variable**	**Male (n = 245)**		**Female (n = 238)**		**Total (N = 483)**		**P value**
	**Mean + SD**		**Mean + SD**		**Mean + SD**		
					**N**	**%**	
Ever had oral sex							0.011
No	223	91.0	230	96.6	453	93.8	
Yes	22	9.0	8	3.4	30	6.2	
Ever had anal sex							0.088
No	227	92.7	229	96.2	455	94.4	
Yes	18	7.3	9	3.8	27	5.6	
Experienced “Open foreskin” (n = 245)							NA
No	70	28.6	Na	Na	70	28.6	
Yes	175	71.4	Na	Na	175	71.4	
Experienced “Vagina breakthrough” (n = 233)**							NA
No	Na	Na	5	2.2	5	2.2	
Yes	Na	Na	228	97.8	228	97.8	
Experienced “Welcome guest” (n = 233)**							NA
No	Na	Na	163	68.5	163	68.5	
Yes			73	31.5	73	31.5	
	mean + sd		mean + sd		mean + sd		
Age at the first time of sexual intercourse (n=411)							< 0.001
11-14 years old	118	48.2	185	77.7	303	62.7	
15-18 years old	127	51.8	53	22.3	180	37.3	
Mean ± SD	14.6	1.46	1.12	13.7	1.38	14.2	
Contraceptive methods used at the first sexual intercourse							<0.001
Condom only	114	46.5	32	13.4	146	30.2	
Condom and other method	25	10.2	14	5.9	39	8.1	
Other method only (OC)	68	27.8	29	12.0	97	20.1	
Not method	38	15.5	163	68.5	201	41.6	
	mean + sd		mean + sd		mean + sd		
Number of sexual partners during their lifetime							< 0.001
1 person	40	16.3	146	61.3	186	38.5	
> = 2 persons	205	83.7	92	38.7	297	61.5	
Mean ± SD	4.4 (4.41)		1.8 (1.95)		3.1 (3.65)		
Sexual intercourse in the last 6 months							< 0.001
No	55	22.4	154	64.7	209	43.3	
Yes	190	77.6	84	35.3	274	56.7	
	mean + sd		mean + sd		mean + sd		
Number of partners in the last 6 months (n = 274)							< 0.001
1 person	87	45.8	58	69.4	145	52.9	
> = 2 persons	103	54.2	26	30.6	129	47.1	
Mean ± SD	2.3 (2.04)		1.5 (1.43)		2.0 (1.90)		
Condom use during the last 6 months							0.642
No	96	50.5	45	53.6	141	51.5	
Yes	94	49.5	39	46.4	133	48.5	
Condom use during the last sexual encounter							0.121
No	103	54.2	37	44.0	140	51.1	
Yes	87	45.8	47	56.0	134	48.9	
How often did you or your partner use a condom when having sexual intercourse during the last six months							0.397
Never	81	42.6	31	36.9	112	40.9	
< half of the occasions	15	7.9	5	6.0	20	7.3	
During half of the occasions	32	16.8	11	13.1	43	15.7	
>half of the occasions	16	8.4	7	8.3	23	8.4	
Always	46	24.2	30	35.7	76	27.7	
Having STIs symptoms during the last year							.034
No	234	95.5	235	98.7	467	97.0	
Yes	11	4.5	3	1.3	14	3.0	

Note:

** = Five female respondents did not answer to questions “Vagina Breakthrough” and “Welcome Guest”.

**NA-**is not applicable as Open foreskin is for boys; “Vagina breakthrough” and “Welcome Guest” are for girls.

Fifty seven per cent of respondents had been sexually active during the last six months prior to the interview. Of those reporting being sexually active in the past six months, 48.5 per cent reported not using a condom (49.5% of sexually active boys and 46.4% of sexually active girls, p = 0.642). Among those who reported using a condom, 27.7 per cent indicated using a condom every time they had sex. There was no significant difference in this response between males and females (24.2% versus 35.7%, p = 0.397). Forty seven respondents (more boys than girls) reported having more than two partners during the last six months prior to the interview with gender difference (54.2% of boys versus 30.6% of girls, p < .001).

Two thirds of girls reported having experiences of “Vagina Breakthrough” and one fifth had experience of “welcome guest”. Nearly 95 per cent of girls initiated Vagina Breakthrough” at age younger than 15 years old and the mean age of Vagina Breakthrough” was 14.7 ± 1.37. For boys, nearly all of them (90.9%) reported having experienced open foreskin. Sixty per cent of boys started open foreskin at age less than 15 and the mean age of open foreskin was 14.2 ± 1.13.

### Knowledge and source of STIs/HIV

The mean score of knowledge on STIs was 3.4 out of 12, suggesting poor knowledge. Almost all respondents were aware that sexual intercourse was the transmission route for STIs. One third were aware that symptoms such as abnormal vaginal discharge and pain during urination were possible indications of STIs. About 26.3 per cent knew about the symptoms of STIs such as abnormal vaginal discharge for women and 36.2 per cent reported about the pain during urination as the STI symptom. Few were aware of possible complications of untreated STIs or that women infected with STIs can be asymptomatic. There was a sex difference regarding knowledge about STIs as female participants had lower knowledge compared to male respondents (60.2% versus 39.8%, p < .001).

Respondents had also a low knowledge about HIV (mean 3.4 ± 2.02 out of 8). Misperceptions included a person will not get HIV if she or he is taking antibiotics (32.5%) and washes genital parts after sex (79.4%). About 40 per cent thought a person could be infected with HIV by sharing a glass of water with someone who was HIV positive. Less than three fifth (55.3%) of them had low knowledge on HIV.

### Psychological construct of self-efficacy and peer sexual norms

The mean score of self-efficacy was 30.5 out of possible score of 65, indicating middle self-efficacy. Males had higher self-efficacy than females (31.6 versus 27.9). Girls had more self-efficacy in relation to being able to abstain than boys (18 versus 17.6), while boys had more self-efficacy in relation to condom use compared to girls (14.4 versus 11.8) (Table 3).

**Table 3:** Score of the self-efficacy and peer sexual norm among sexually experienced respondents

**Statement**	**Total (483)**	**Male (245)**	**Female (238)**
	**Mean (SD)**	**Mean (SD)**	**Mean (SD)**
	**Min: max**	**Min: max**	**Min: max**
Self-Efficacy in abstinence	17.8 (9.264)	17.6 (8.930)	18.0 (9.604)
	0: 40	0: 39	0: 40
Self efficacy in condom use	13.6 9 (6.533)	14.4 (6.491)	11.8 (6.307)
	0: 28	0: 28	0: 27
Self-efficacy in partner communication	3.8 (2.259)	3.9 (2.189)	3.6 (2.409)
	0: 8	0: 8	0: 8
Total mean score self-efficacy	30.5 (12.475)	31.6 (12.771)	27.9 (11.438)
	0: 65	0: 65	5: 64
Peer sexual norms	5.4 (3.028)	5.7 (3.095)	5.1 (2.931)
	0 & 14	0 & 13	0 & 14

Most of respondents reported permissive peer sexual norms with a mean of 5.4 of 14. Females had fewer peers with permissive sexual norms than male respondents (5.1 + 2.93 versus 5.7 + 3.09).

### Reported STIs symptoms

Only 4.1 per cent respondents reported having STI symptoms during the six months preceding the interview (Data not shown). Among those respondents reported having had STI symptoms, the most common STI symptoms was painful/burning sensation when urinating (30%), itching around the sex organ (30%) and discharge (25%). Males were more likely to report STI symptoms compared to female respondents (6.9% versus 1.3%, p = .002). Three per cent of respondents reported a history of symptoms or signs associated with an STI during the past year, but there was no gender difference in reporting having STIs symptoms STIs (4.5% versus 1.3%, p = .034) (Table 2).

### Perception of risk of STIs and HIV

A high percentage of respondents reported that they were very afraid of getting STIs (82.6%) and HIV (85.7%) while fewer reported that they were a little afraid (12.4% and 11.6%) of getting STI or HIV (Table 4).

**Table 4:** Perception of risk getting STIs and HIV among sexually experienced adolescents (N=483)

**Perception**	**Male (n = 245)**		**Female (n = 238)**		**Total (n = 483)**		**P value**
	**N**	**%**	**n**	**%**	**N**	**%**	
**Afraid of getting STIs**							0.362
Not afraid at all	20	8.2	27	11.3	47	9.7	
Afraid a little	8	3.3	5	2.1	13	2.7	
Afraid somewhat	15	6.1	9	3.8	24	4.9	
Afraid a lot	202	82.4	197	82.8	399	82.1	
**Perception of getting STIs**							0.001
No risk	154	62.9	124	52.1	278	57.2	
Low risk	49	20.0	34	14.3	83	17.1	
Medium risk	22	9.0	41	17.2	63	13	
High risk	20	8.2	39	16.4	59	12.1	
**Afraid of getting HIV/AIDS**							0.581
Afraid at all	21	8.6	25	10.5	46	9.5	
Afraid a little	4	1.6	6	2.5	10	2.1	
Afraid somewhat	5	2.0	8	3.4	13	2.7	
Afraid a lot	215	87.8	199	83.6	414	85.2	
**Perception of getting HIV/AIDS**							0.004
No risk	155	63.3	127	53.4	282	58.4	
Low risk	46	18.8	35	14.7	81	16.8	
Medium risk	24	9.8	37	15.5	61	12.6	
High risk	20	8.2	39	16.4	59	12.2	

Twelve per cent of respondents considered themselves to be at high risk for getting STIs and 13 per cent regarded themselves to be at medium risk. An additional 17.2 per cent of the participants considered themselves to have low risk while 57.6 per cent regarded themselves to have no risk at all. Similar pattern of perceived risk to HIV was found with 58.4 per cent considering themselves to be at no risk. There was a statistically significant sex difference of the perception of risk of STIs and HIV with more females reporting to have a high risk compared to males.

### Correlates of risk perception

In bivariate analysis, level of education, knowledge about sexual transmission of STIs, peer sexual norms, early age at first sex, multiple sexual partners, and exposure to STI symptoms were significant related to adolescent’s perception of personal risk of STIs (see Table 5). While male sex, knowledge of STIs and HIV, peer sexual norms, early age at first sex, and multiple sex partners were significantly associated with perceived risk of HIV (Table 6).

**Table 5:** Bivariate and multivariate ordinal analyses of factor associated with risk perceptions of getting STIs among sexually experienced adolescents in LNT province (N = 483)

**Factors**	**No risk %**	**Low risk %**	**Medium risk %**	**High risk %**	**Crude**		**Adjusted**	
					**OR**	**95% CI**	**OR**	**95% CI**
Sex (n = 483)								
Female	52.1	14.3	17.2	16.4	1		1	
Male	62.9	20.0	9.0	8.1	0.6**	0.4 – 0.8	0.5*	0.3 – 0.8
Age								
14–15 yrs	55.0	17.0	12.6	15.4	1		1	
16–19 yrs	59.1	17.3	13.3	10.3	0.8	0.6-1.1	0.8	0.5-1.2
Ethnicity								
Non-Akha	52.0	25.3	13.3	9.4	1		1	
Akha	58.6	15.7	13.0	12.7	0.9	0.6-1.4	0.5	0.3-0.9
Level of education								
Never go to school	52.3	15.0	15.9	16.8	1		1	
Primary school	57.5	17.6	11.2	13.7	0.8	0.5-1.2	0.8	0.5-1.4
Secondary school & higher	61.5	18.2	14.0	6.3	0.6*	0.4-0.98	0.7	0.4-1.2
Living arrangement							NA	
Family	57.0	17.2	13.4	12.4	1			
Others	76.9	15.4	0	7.7	0.4	0.1-1.4		
Knowledge on STIs								
Mean (SD)	3.0 (1.44)	3.8 (1.91)	4.4 (1.77)	3.5 (1.26)				
Min: Max	0: 9	0: 9	1: 10	1: 6	1.3***	1.2-1.5	1.3***	1.1-1.4
Knowledge on HIV/AIDS								
Mean (SD)	3.2 (1.91)	4.4 (2.17)	3.6 (1.95)	2.8 (1.60)				
Min: Max	0: 8	0: 8	0: 8	0: 8	1.1	0.97-1.2	NA	
Self-efficacy								
Mean (SD)	29.3 (12.67)	33.2 (12.08)	31.6 (10.21)	30.2 (14.04)				
Min: Max	0: 65	8: 64	11: 55	4: 50	1.01	0.99-1.03	NA	
Peer sexual norms								
Mean (SD)	5.6 (3.14)	5.7 (2.78)	4.4 (2.70)	5.0 (2.93)				
Min: Max	0: 14	0: 12	0: 12	0: 12	0.9*	0.87-0.98	0.9	0.9-1.1
Early age at first sex								
<15 years	54.1	17.5	13.5	14.9	1		1	
>15 years	63.3	16.7	12.2	7.8	0.7*	0.5-0.9	0.9	0.6-1.3
Multiple sex partners								
1 person	51.1	15.1	16.1	17.7	1		1	
> = 2 persons	61.6	18.5	11.1	8.8	0.6*	0.4-0.8	0.8	0.5-1.3
Custom sexual practice							NA	
No	50.0	25.0	16.3	8.7	1			
Yes	59.1	15.6	12.4	12.9	0.8	0.5-1.3		
Anal sex							NA	
No	57.7	17.3	12.9	12.1	1			
Yes	55.6	14.8	14.8	14.8	1.1	0.5-2.4		
Condom use during the last 6 months								
No	60.3	22.0	9.2	8.5	1		NA	
Yes	55.6	19.6	13.5	11.3	1.3	0.8-2.0		
Having STIs symptoms								
No	58.3	17.3	13.4	11/0	1		1	
Yes	40.0	15.0	5.0	40.0	3.0*	1.2-7.2	3.1*	1.1-9.2

*P < 0.05.

**P < 0.005.

***P < 0.001.

**Table 6:** Bivariate and multivariate analyses of factor associated with risk perceptions of getting HIV among sexually experienced adolescents in LNT province

**Factors**	**No risk %**	**Low risk %**	**Medium risk %**	**High risk %**	**Crude**		**Adjusted**	
					**OR**	**95% CI**	**OR**	**95% CI**
Sex								
Female	53.4	14.7	15.5	16.4	1		1	
Male	63.3	18.8	9.8	8.2	0.6**	0.4-0.8	0.5**	0.3-0.8
Age								
14–15 yrs	53.3	18.1	13.7	14.8	1		1	
16–19 yrs	61.5	15.9	12.0	10.6	0.7	0.5-1.01	0.7	0.5-1.2
Ethnicity								
Non-Akha	53.3	22.7	14.7	9.3	1		1	
Akha	59.3	15.7	12.3	12.7	0.9	0.6-1.4	0.6	0.3-1.1
Level of education								
Never go to school	53.3	17.7	10.3	18.7	1		1	
Primary school	59.6	16.3	12.9	11.2	0.7	0.5-1.1	0.8	0.5-1.3
Secondary school & higher	60.1	16.8	14.0	9.1	0.7	0.4-1.1	0.7	0.4-1.3
Living arrangement								
Family	58.5	16.6	12.6	12.3	1		NA	
Others	53.8	23.1	15.4	7.7	1.1	0.4-2.9		
Knowledge on STIs								
Mean (SD)	3.0 (1.40)	4.0 (1.94)	4.0 (1.85)	3.7 (1.40)				
Min: Max	0: 9	1: 9	0: 10	1: 7	1.3***	1.2-1.5	1.3***	1.2-1.5
Knowledge on HIV /AIDS								
Mean (SD)	3.2 (1.96)	4.3 (2.13)	3.6 (1.68)	3.1 (1.86)				
Min: Max	0: 8	0: 8	0: 8	0: 8	1.1*	1.0-1.2	1.1*	1.1-1.4
Self-efficacy								
Mean (SD)	29.1 (12.95)	33.9 (11.27)	31.4 (10.94)	31.2 (12.48)				
Min: Max	0: 65	8: 64	10: 56	4 50	1.01	0.99-1.03	NA	
Peer sexual norms								
Mean (SD)	5.7 (3.12)	5.0 (2.75)	4.6 (2.78)	5.2				
Min: Max	0: 14	0: 13	0: 12	(2.99) 0: 12	0.9*	0.86-0.97	0.95	0.89-1.02
Early age at first sex								
<15 years	54.5	17.8	11.9	15.8	1		1	
>15 years	65.0	15.0	13.9	6.1	0.6*	0.4-0.9	0.9	0.6-1.5
Multiple sex partners								
1 person	52.1	16.1	16.7	15.1	1		1	
> = 2 persons	62.3	17.2	10.1	10.4	0.6*	0.4-0.9	0.95	0.6-1.5
Custom sexual practice								
No	53.8	21.2	16.2	8.8	1		NA	
Yes	59.3	15.9	11.9	12.9	0.9	0.6-1.4		
Anal sex								
No	57.7	16.9	12.9	12.5	1		NA	
Yes	70.4	14.8	7.4	7.4	0.6	0.2-1.3		
Condom use during the last 6 months								
No	59.6	21.3	7.8	11.3	1		NA	
Yes	57.1	13.5	14.3	15.1	1.2	0.8-2.0		
Having STIs symptoms							NA	
No	58.5	16.9	12.7	11.9	1			
Yes	55.0	15.0	10.0	20.0	1.3	0.5-3.0		

*P < 0.05.

**P < 0.005.

***P < 0.001.

After controlling for confounding variables, in multivariate logistic regression analysis, factors associated with perceived personal high risk of getting STIs were: being male (OR = 0.5, 95% CI: 0.3-0.8, p ≤ .05), high level of knowledge about STIs (OR = 1.3, 95% CI: 1.1-1.4, p ≤ .001); and having had symptoms of STIs in last six months (OR = 3.1, 95% CI: 1.1-9.2, p ≤ .05) (Table 5). Perceived risk of getting HIV was significantly associated with being male (OR = 0.5, 95% CI: 0.3-0.8, *p* ≤ 0.01), having more knowledge about STIs (OR = 1.3, 95% CI: 1.2-1.5, *p* ≤ .001), and knowledge about HIV (*OR* = 1.1, 95% CI: 1.1-1.4, *p* ≤ .05) (Table 6).

## Discussion

This study explores risk perceptions regarding STIs and HIV and associated factors across a wide spectrum of socio-demographic and socio-cultural context in Northern Lao PDR. Our results indicate that the individual and psychological factors are important in understanding risk perception of getting STIs and HIV.

### Risk perception of STIs/HIV

The study revealed a tendency for adolescents, especially boys, to underreport their risk of contracting HIV. Slightly higher than two thirds of our respondents (74.4% and 75.2%) perceived themselves as to be at no or low risk of STIs and HIV infection respectively even though they reported that they had unprotected sex, which could result in STIs/HIV infection. This could be a reflection of their low level of knowledge about STIs and HIV and the low knowledge of what should be considered risk factors for STI and HIV. Similarly, previous study carried out in Luangnamtha province revealed an alarming lack of HIV/AIDS knowledge [47]. Misconceptions and erroneous views about HIV transmission may influence decisions and also make the person interpret the actual odds of getting an infection wrongly [48,49], which vary across cultural context. The other reason could be that the participants did not know how to assess their risk and they might not be able to apply their knowledge of disease transmission to assess their risk level every time they engage in sexual activity. Furthermore fewer girls than boys reported that they were at risk compared to boys. These findings highlight the gap between the level of risk perception versus the actual sexual risk behaviors and also show a lack of understanding regarding the consequences of risky sexual behaviors. These findings were consistent with previous studies carried out in elsewhere [50,51], but there was no data on the risk perception to HIV/AIDS in Lao PDR to compare.

### Predictors of perceived risk getting of STIs/HIV

Existing literature show mixed results on the association between risk perception and sexual behavior. Our findings suggest that perception of the risk of getting STIs/HIV among adolescents in Luangnamtha province was related to a number of factors operating at the individual level. Demographic factors that influence risk perception are sex, knowledge about STIs and HIV, and peer sexual norms also being strongly correlated with the risk judgments of individual perception regarding risk.

The influence of gender has been well documented, with boys tending to judge risks as smaller as and less problematic than girls [52-54]. Explanations have focused mainly on biological and social factors. For example, it has been suggested that girls are more socialized to care for human health and are less likely to be familiar with science and technology [55]. Other issues relate to power imbalance and social control [56]. In Lao PDR for example, men typically feel more empowered to express their sexual desires and sexually risky behaviour among male is generally more acceptable [9,47]. This could be also explained that women and men often express different levels of concern about the same risks; they perceive different risks and different meaning attributed to the same risks [51].

The study found no association between the traditional sexual customs and perceived risks of STIs/HIV, which was similar to previous studies [19]. The explanation could be that the traditional sexual practices may not necessarily lead to increased perception of HIV risk if these practices are socially supported by the community and families [10]. For Akha adolescents for example, the perceived risk of STIs/HIV is likely to be interpreted within their cultural beliefs in which practices such as Breakthrough Vagina are an essential and normal part of transitioning into a mature adult [9].

### Knowledge about STIs and HIV

The association between perceiving oneself to be at high risk and having knowledge about STIs/HIV is plausible. Respondents who had high knowledge about STIs/HIV might have felt more at risk because they knew that they might get infected. Those unaware of this on the other hand, might believe that only those looking sick, in practice terminally ill persons, might pose a risk for transmission, which is a false and potentially dangerous perception. They might apply their knowledge of disease transmission to assess their risk level every time they engage in sexual activity. This is supported by the fact that women had less knowledge and also fewer women report that they were at risk. Our study was similar to previous studies [51,57] which suggested a positive association between risk perception of STIs/HIV and knowledge about STIs/HIV. The HBM considers knowledge about AIDS as essential for sexual behavior change. For instance, some researchers Zellner [58] described knowledge about AIDS as a form of self-empowerment that may influence a person’s perception of risk and create awareness and thus stimulate behaviour change [58]. On the other hand, some researchers have found that having more knowledge about HIV did not correlate significantly with risk perception for HIV [59]. However, there are no previous studies related to risk perceptions have been carried out in the Lao PDR, highlighting the need to have valid and comparable data.

In our study, risk perception related to STIs and HIV was not associated with number of sex partners during lifetime, condom use and age at first sex. The reason could be that the participants did not perceive themselves at risk or they did not perceive themselves at risk because they have stable sexual relationships or they were not aware of their partner’s sexual practice. The lack of significant association between condom use and perceived risk of getting STIs and HIV might reflect the low preference for condom use relative to other methods such as abstinence. On the other hand, other studies from sub-Saharan Africa have found a positive association between perceived risk and risky sexual behaviour for both women and men [19,60]. Some researchers found a negative association between perceived risks of HIV with sexual behaviours [22,23]. Further the cultural acceptability of multiple partners, particularly for males [9], may moderate young people’s perception of risk.

### Reported STIs symptoms

Having STIs symptoms reinforce adolescent’s STIs and HIV/AIDS risk perception [18]. Our study found a positive association between reporting having symptoms of STIs and perceived risk of getting STIs/HIV, which is similar to previous studies [61]. This could be explained by the fact that those who had STIs had become aware that they were not immune to diseases, including HIV/AIDS. It could also be an effect of the STI/HIV educational campaign program from the Ministry of Health, Lao PDR. Having an STI symptom might lead adolescents to get to know prevention measure of STIs and HIV. This knowledge facilitates adolescent’s awareness and might assist them to identify risky situations. However, some researchers found no association between reporting of STIs and perceived of risk getting STIs [62,63]. In Lao PDR, to date there are no studies on the relationship of reported STIs symptoms and risk perception of STIs/HIV to compare.

### Limitation

This study must acknowledge some limitations. Foremost, any generalization from the results of this study should be made with caution. Because of the cross-sectional nature of our data we are not able to draw conclusions about causal effect. Additional studies using a prospective cohort design will be necessary to evaluate the significance and stability of these predictors to influence perceived risk of getting STIs/HIV/AIDS. Secondly, we over sampled Akha ethnicity aged 14-19 years old, which is due to concentration of the ethnic group Akha in the Long and Sing districts and high proportion of sexually active Akha adolescents compared to adolescents from other ethnic groups. The level of risk in this sample is likely to be higher than among adolescents from other ethnic groups due to traditional sexual customs. This may limit the reliability of the findings, especially as they relate to ethnic differences. Thirdly, we need to acknowledge the difficulties in measuring knowledge and other psychological constructs, which did not demonstrate strong psychometric properties in our sample, probably because of the low literacy level. This indicates a need to refine these measures and be cautious when interpreting the findings. The measurement of risk perceptions by using a single item is not reliable compared to scale construct. However, many previous studies have used single-item variables rather than scales [64,65]. The sensitive nature of sexual behaviors such as sexual practice, number of sexual partners, condom use, and past STIs might have been underreported because of social desirability bias. A number of steps however were undertaken to minimize this, including use of trained interviewers, gender matching between interviewers and participants, and ensuring confidential interview settings. Despite these limitations the study contributes to an understanding of the risk perceptions of an under-researched population in relation to their vulnerabilities to STIs/HIV infection. The findings are useful for informing interventions that aim to increase young people’s ability to accurately gauge their risks relative to their sexual behaviour. More qualitative research will provide further understanding of the link between risk perceptions, risk taking and factor shaping perceived risk of getting STIs and HIV/AIDS among young people require that attention is paid to the dynamics of sexual relationship, the individual characteristics, and the structural environmental factors that facilitate preventive behaviors.

## Conclusion

This study investigated the risk perceptions and factors associated with the risk perception of getting STIs/HIV among adolescents in one rural area of Lao PDR. Socio-demographic factors, knowledge of STIs/HIV, and the level of exposure to STIs were the main determinants of the risk perception of STIs/HIV.

These findings highlight a need for programmes that can create greater awareness of the risk of STIs/HIV among young adolescents in the Northern part of Lao PDR. Young people should be at the center of strategies to control HIV infection and addressing inaccurate perception of risk may be a key to improve safer sexual practices. The risk of STIs and HIV/AIDS infection is higher for unprotected anal sex compared with unprotected vaginal and oral sexual intercourse. Tailored messages are therefore needed to address adolescent risk perceptions related to anal sex as well as other forms of sexual intercourse. This study also underscores the complexity of the relationship between risk perception and sexual risk behaviors and traditional cultural practices thus the need to study the relationships between sexual risk behaviors and perception of risk behaviors more in depth. More qualitative research is needed to explore the structural and environmental circumstances that characterize risk perception and sexual relations, the social dimension of risk, the reasons of risk, and the life skills needed in order to negotiate risk reduction.

## Competing interests

The authors declare that they have no competing interests.

## Authors’ contributions

SV developed the research proposal, designed the instrument, and collected data in the field sites, analyzed and wrote the draft manuscript. EF contributed to the development of research design, analyzing and finalized the manuscript. KC assisted in the survey instrument development, data collection, data analysis and also contributed to the final version of the manuscript. ST contributed to the analysis and writing manuscript. All authors read and approved the final manuscript.

## Pre-publication history

The pre-publication history for this paper can be accessed here:

http://www.biomedcentral.com/1471-2458/13/1126/prepub

## Supplementary Material

Additional file 1 Risk perceptions of STIs/HIV and sexual risk behaviours among sexually experienced adolescents in the Northern part of Lao PDR. Click here for file
