# Supplementary material for: Risk perceptions of STIs/HIV and sexual risk behaviours among sexually experienced adolescents in the Northern part of Lao PDR
Source: BMC Public Health. 2013 Dec 5;13:1126. doi: 10.1186/1471-2458-13-1126 (PMC3890592; doi:10.1186/1471-2458-13-1126)
Supplement: Additional file 1 — Risk perceptions of STIs/HIV and sexual risk behaviours among sexually experienced adolescents in the Northern part of Lao PDR. [file 1471-2458-13-1126-S1.doc]

Questionnaire for the study entitled “**Risk perceptions of STIs/HIV and Sexual Risk behaviours among sexually experienced adolescents in the Northern part of Lao PDR ”**

I. Socio-demographic data

| Questions | Coding categories | | Skip to |
| --- | --- | --- | --- |
| 1.1. Sex of respondent | Male Female | 1  2 |  |
| 1.2. Date of birth | __/__/__ |  |  |
| 1.3. How old are you when at last  birthday? | __ age in years |  |  |
| 1.4. What is the highest level of schooling  you completed? | Never go to school  Primary  Secondary  High school  Technical (primary level)  Technical (middle level)  Technical (high level)  University  Other, (specify) _____________ | 1  2  3  4  5  6  7  8  9 |  |
| 1.5. Have you ever worked for pay? | Yes  No | 1  0 | 1.12 |
| 1.6. How old were you when you start  working? | _ _ age in years |  |  |
| 1.7. Are you currently working for pay? | Yes  No | 1  0 | 1.12 |
| 1.8. What type of work did you do? | Not working/Unemployed  Farmer or Gardener  Government Civil service  Private Civil service  Unskilled worker (daily basis)  Skilled worker  Other,___________________ 7 | 1  2  3  4  5  6  7 |  |
| 1.9. How much do you earn in a month? | _ _ _ _ _ _ _ Kips |  |  |
| 1.10. What are the source of your  income?  (Circle all that apply) | Working  Parents  Scholarship  Relatives Others................... | 1  2  3  4  5 |  |
| 1.11. Is your income enough for your  expenses? | Yes  No | 1  0 |  |
| 1.12. What is your birth order? | ______________Order |  |  |
| 1.13. Where do you live? | Live with family/relative  Dormitory  Rent a house  Other.............................................. | 1  2  3  4 |  |
| 1.14. Did you experienced menstruation/male  Puberty changes | Yes  No | 1  0 |  |
| 1.15. Age of menarche for girls | -------- years |  |  |
| 1.16. Experienced vagina breakthrough  (For girls) | Yes  No | 1  0 |  |
| 1.17. Age experience vagina breakthrough | -------- years |  |  |
| 1.18. Experienced open foreskin  (For boys) | Yes  No | 1  0 |  |
| 1.19. Age experience open foreskin  (For boys) | -------- years |  |  |
| 1.20. Experienced of welcome guest | Yes  No | 1  0 |  |
| 1.21. Age experience of welcome guest | -------- years |  |  |

**II. Family Characteristic**

|  | Questions | Coding categories |  | Skip to |
| --- | --- | --- | --- | --- |
| 2.1 | What is the marital status of your parent? | Incohabating/Single  Married  Separated  Widowed/divorced | 1  2  3  4 |  |
| 2.2 | Is your father alive? | Yes  No | 1  0 | 2.15 |
| 2.3 | Does he live with the same house with you? | Yes  No | 1  0 |  |
| 2.4 | What is the age of your father? | _ _ in years |  |  |
| 2.5 | What is the highest level of education that your father completed? | Primary  Secondary  Technical  University  Postgraduate | 1  2  3  4  5 |  |
| 2.6 | Is your father currently working? | Yes  No | 1  0 | 2. 8 |
| 2.7 | .What is your father’s occupation? | Government Officer  Private Officer  Commercial/Business  Farmer/agriculture  Others……. | 1  2  3  4  5 |  |
| 2.8 | Is your mother alive? | Yes  No | 1  0 | 2.15 |
| 2.10 | Does she live with the same house  with you? | Yes  No | 1  0 |  |
| 2.11 | What is the age of your mother? | _ _ in years |  |  |
| 2.12 | What is the highest level of education that your mother completed? | Primary  Secondary  Technical  University  Postgraduate | 1  2  3  4  5 |  |
| 2.13 | Is your mother currently working? | Yes  No | 1  0 | 2.15 |
| 2.14 | What is your mother’s occupation? | Government Officer  Private Officer  Commercial/Business  Farmer/agriculture  Others……. | 1  2  3  4  5 |  |
| 2.15 | What is your family size? | ______________ |  |  |
| 2.16 | What is your family structure? | Parent & children  Mother & children  Father & children  Grandparents, parents & children | 1  2  3  4 |  |

**III. Knowledge of reproductive and pregnancy**

|  | Questions | Category answers | Code | Skip to |
| --- | --- | --- | --- | --- |
| 3.1 | Time during menses that women can get pregnancy | Right before menses begins  During the period  About a week after period  About 2 weeks after period  No making difference  All times | 1  2  3  4  5  6 |  |
| 3.2 | Woman could fall pregnant during the first sexual intercourse | Yes  No | 1  0 |  |
| 3.3 | Unprotected vaginal sexual intercourse can lead to pregnancy, STIs/HIV/AIDS | Yes  No | 1  0 |  |
| 3.4 | Hormones can affect body shape and size, body hair growth and other changes | Yes  No | 1  0 |  |
| 3.5 | Kissing/hugging does not result in pregnancy | Yes  No | 1  0 |  |

**IV. Knowledge of contraception and** accessibility to contraceptives

|  | Questions | Category answer | Code | Skip to |
| --- | --- | --- | --- | --- |
| 4.1 | Did you hear any contraceptive method? | Yes  No | 1  0 | Go to 6.3 |
| 4.2 | From where, have you ever heard about contraceptives?  Multiple answer | School  Parents  Friends  Peer educator from projects  Media  Health staff  Other | 1  2  3  4  5  6  7 |  |
| 4.3 | Which method did you heard? | Pill  IUD  Injection  Diaphragm  Condom  Female sterilization  Male sterilization  Rhythm/periodic abstinence  Withdrawal  Norplant  Traditional  Emergency method  Other | 1  2  3  4  5  6  7  8  9  10  11  12  13 |  |
| 4.4 | Whit whom are you talking about family planning?  Multiple answer | Never talk  Wife  Parent  Brother/Sister  Son/Daughter  Relative  Friend  Other | 1  2  3  4  5  6  7  8 |  |
| 4.5 | The conception could take place if they had missed taking their contraceptive pill once | Yes  No | 1  0 |  |
| 4.6 | Condom use prevented sexually transmitted diseases | Yes  No | 1  0 |  |
| 4.7 | Condom use prevented both sexually transmitted diseases and pregnancy (dual methods) | Yes  No | 1  0 |  |
| 4.8 | A condom could be used only once. | Yes  No | 1  0 |  |
| 4.9 | Condoms do not slip off the man during intercourse and disappear inside a woman’s body | Yes  No | 1  0 |  |
| 4.10 | Did you ever used contraceptive | Yes  No | 1  0 |  |
| 4.11 | If yes, Where did you get contraceptives? | Pharmacy  Health center  District/Provincial Hospital  VHV  Friends  Bought in the market  Other…. | 1  2  3  4  5  6  7 |  |
| 4.12 | Why did you choose that place as the first one for treatment? (*can choose many*) | Confidentiality,  Convenient  Skilled provider  Friendly provider  Cheap/Free of charge Recommended by friends/ relatives  Good equipments  Service available as desired  Prefer female examiner  Other | 1  2  3  4  5  6  7  8  9  10 |  |
| 4.13 | Barriers of accessing contraceptive method for adolescents | No barrier  Not knowing where to go  Inconvenient hours  Privacy not respected  Not treated nicely by staff  Costly, not able to pay  No same sex provider  Afraid or fearful  Embarrassed or shy  Too young  Other | 1  2  3  4  5  6  7  8  9  10  11 |  |
| 4.14 | Do you use FP / contraception currently? | Yes  No | 1  0 |  |
| 4.15 | If yes, which method do you use? | Pill  IUD  Injection  Diaphragm  Condom  Female sterilization  Male sterilization  Rhythm/periodic abstinence  Withdrawal  Norplant  Traditional  Emergency method  Other | 1  2  3  4  5  6  7  8  9  10  11  12  13 |  |
| 4.16 | What are the methods of contraception that are easily accessible your local health care facilities?  (Multiple responses) | Pill  IUD  Injection  Diaphragm  Condom  Female sterilization  Male sterilization  Rhythm/periodic abstinence  Withdrawal  Norplant  Traditional  Emergency method  Other | 1  2  3  4  5  6  7  8  9  10  11  12  13 |  |

**V**. Knowledge of STIs

|  | **Questions** | | **Category answers** | **Coding** | | |  |
| --- | --- | --- | --- | --- | --- | --- | --- |
| **5.1** | Have you Heard or seen messages related to STIs? | Yes  No | | | 1  0 |  | |
| **5.2** | From which sources of information have you heard about it? Multiple answers | School  Parents  Friends  Peer educator from projects  Media  Health staff  Other | | | 1  2  3  4  5  6  7 |  | |
| **5.3** | Do you know what are considered suspected symptoms of STIs ?  (Multiple answer) | Abnormal vaginal discharge (female)  Urethral discharge (male)  Genital ulcers  Genital warts  Genital itching  Pain during urination  Pain during sexual intercourse  Lower abdominal pain (female)  Don't know  Other (specify)…........……… | | | 1  2  3  4  5  6  7  8  9  10 |  | |
| **5.4** | Can STIs transmit? | Yes  No  DK | | | 1  0  9 |  | |
| **5.5** | *If yes, what are routes of transmission?* (Multiple answer) | Sexual intercourse  Blood transfusion  Sharing needle  Mother to child  Sharing clothes, things  Don't know  Other *(specify)*………………… | | | 1  2  3  4  5  9  6 |  | |
| **5.6** | Is it necessary to treat husband/wife /partner of STI patients'? | Yes  No  DK | | | 1  0  9 |  | |
| **5.7** | In your opinion, can STIs be cured? | Yes  No  DK | | | 1  0  9 |  | |
| **5.8** | What are complications of STIs if untreated?  (Multiple answers) | Infertility  Ectopic pregnancy.  Cervical cancer  Premature birth  Still birth  Neonatal death  Do not know  Other (specify)............................. | | | 1  2  3  4  5  6  7  9 |  | |
| **5.9** | Can STIs be preventable? | Yes  No  DK | | | 1  0  9 |  | |

**VI**. Knowledge of HIV/AIDS

|  | Questions | Coding categories | | |
| --- | --- | --- | --- | --- |
|  |  | Y | N | DK |
| 6.1 | Have you Heard or seen messages related to HIV/AIDS |  |  |  |
| 6.2 | From which sources of information have you heard about it? Multiple answers | School  Parents  Friends  Peer educator from projects  Media  Health staff  Other | 1  2  3  4  5  6  7 |  |
| 6.3 | A person can get HIV by sharing a glass of water with someone who has HIV |  |  |  |
| 6.4 | Pulling out the penis before a man climaxes/cums keeps a woman from getting HIV during sex |  |  |  |
| 6.5 | Washing genital parts after sex keeps a person from getting HIV |  |  |  |
| 6.6 | All pregnant women infect HIV will have babies born with AIDS |  |  |  |
| 6.7 | People who have been HIV quickly show serious signs of being infected |  |  |  |
| 6.8 | People are likely to get HIV by deep kissing, putting their tongue in their partner’s mouth, if their partner has HIV |  |  |  |
| 6.9 | A woman cannot get HIV if she has sex during her period |  |  |  |
| 6.10 | There is female condom that can decrease a women’s chance of getting HIV |  |  |  |
| 6.11 | A person will not get HIV if she or he is taking antibiotic |  |  |  |
| 6.12 | Having sex more than one partner can increase a person’s chance of being infected with HIV |  |  |  |
| 6.13 | Taking a test for HIV one week after having sex will tell a person if she or he has HIV |  |  |  |
| 6.14 | A person can get HIV from oral sex |  |  |  |

**VII. Attitudes towards sexual activities and sex education**

|  | Questions | Categories | Code | Skip |
| --- | --- | --- | --- | --- |
|  | **Attitudes towards sexualities** |  |  |  |
| 7.1 | Young unmarried women should not have sex until they marry | Strongly disagree  Disagree  Agree  Strongly agree | 1  2  3  4 |  |
| 7.2 | Young unmarried men should not have sex until they marry | Strongly disagree  Disagree  Agree  Strongly agree | 1  2  3  4 |  |
| 7.3 | Unmarried young people  should not even have  opposite-sex friendships | Strongly disagree  Disagree  Agree  Strongly agree | 1  2  3  4 |  |
| 7.4 | Sexual relationships between  unmarried young people are  not acceptable even when  they love each other | Strongly disagree  Disagree  Agree  Strongly agree | 1  2  3  4 |  |
| 7.5 | Young people who have premarital sex should be condemned for their low social morals; | Strongly disagree  Disagree  Agree  Strongly agree | 1  2  3  4 |  |
| 7.6 | Young people who have premarital sex should be punished | Strongly disagree  Disagree  Agree  Strongly agree | 1  2  3  4 |  |
|  | **Attitudes towards sex education/peer educators** |  |  |  |
| 7.7 | It is important for sex education to be taught in schools | Strongly disagree  Disagree  Agree  Strongly agree | 1  2  3  4 |  |
| 7.8 | It is important for sex education to be taught among out-of-school youth | Strongly disagree  Disagree  Agree  Strongly agree | 1  2  3  4 |  |
| 7.9 | Discussing sex education with young people encourages young people to have sex | Strongly disagree  Disagree  Agree  Strongly agree | 1  2  3  4 |  |
| 7.10 | Youth 12–14 years old should be taught about how to avoid AIDS | Strongly disagree  Disagree  Agree  Strongly agree | 1  2  3  4 |  |
| 7.11 | Youth 12–14 years old should be taught about using a condom to avoid AIDS | Strongly disagree  Disagree  Agree  Strongly agree | 1  2  3  4 |  |

**VIII. Sexual Behavior and condom use**

| **No** | **Sexual intercourse** | **Category answers** | **Code** | **Skip** |
| --- | --- | --- | --- | --- |
| 8.1 | Have you ever had any sexual  intercourse?  [By sexual intercourse, I mean vaginal sex (penis and vagina), oral, anal sex] | Yes  No | 1  0 | Q 8.1 |
| 8.2 | Have you ever had vaginal sexual intercourse? | Yes  No | 1  0 |  |
| 8.3 | Have you ever had oral sex? | Yes  No | 1  0 |  |
| 8.4 | Have you ever had anal sex? | Yes  No | 1  0 |  |
| 8.5 | How old were you at the first time of sexual intercourse? | _ _ age in years |  |  |
| 8.6 | In your lifetime, with how many different partners have you had sexual intercourse? | _ _ No of partners |  |  |
| 8.7 | In the last 6 months, have you ever had any sexual intercourse? | Yes  No | 1  0 | Next Section |
| 8.8 | In the last 6 months, how many partners have you had? | _ _ No of partners |  |  |
| 8.9 | Have you ever had sex without a condom during the last 6 months? | Yes  No | 1  0 |  |
| 8.10 | Did you use a condom during this most recent sexual encounter? | Yes  No | 1  0 |  |
| 8.11 | At the beginning of the relationship with your current partner, how often did you or your partner use a condom when having sexual intercourse? | Never  < half the time  half the time  > half the time  Always  Refused | 0  1  2  3  4  8 |  |

(c) **In the last 6 months,** have you ever had sex with the following partners? If so,

how often did you use a condom? Have you been high on rug use or drunk?

| **No** | **Have you had sexual intercourse with_[A]_** | **How many**  **Partner** | **How often did you or your partner use a condom?** |  | **When you had sexual intercourse with _[A]_, have you ever been high on drugs?** | **When you had sexual intercourse with _[A]_, have you ever been drunk?** |
| --- | --- | --- | --- | --- | --- | --- |
| 8.12 | Girlfriend or  Boyfriend  Yes 1  No 0 | __ __ | Never  < half the time  half the time  > half the time  Always  Refused | 0  1  2  3  4  5 | Yes 1  No 0  DK 7 | Yes 1  No 0  DK 7 |
| 8.13 | Casual partner  Yes 1  No 0 | __ __ | Never  < half the time  half the time  > half the time  Always  Refused | 0  1  2  3  4  5 | Yes 1  No 0  DK 7 | Yes 1  No 0  DK 7 |
| 8.14 | Male/ female sex workers  Yes 1  No 0 | __ __ | Never  < half the time  half the time  > half the time  Always  Refused | 0  1  2  3  4  5 | Yes 1  No 0  DK 7 | Yes 1  No 0  DK 7 |
| 8.15 | Had sex in exchange for money  Yes 1  No 0 | __ __ | Never  < half the time  half the time  > half the time  Always  Refused | 0  1  2  3  4  5 | Yes 1  No 0  DK 7 | Yes 1  No 0  DK 7 |

**IX.** Self-efficacy

| **No** | | **Self-efficacy** | | **Category answers** | | **Code** | | **Skip** | |  |
| --- | --- | --- | --- | --- | --- | --- | --- | --- | --- | --- |
|  |  | | **Self-efficacy in abstinence** | | | | | | | |
|  | 9.1 | | How much do you feel confidence that you do not want to have intercourse? | | Not at all  A little  Slightly  Somewhat  Very likely | | 0  1  2  3  4 | |  | |
|  | 9.2 | | How much do you feel confidence that you are able  to refuse having sex with a person they had known for only a few days? | | Not at all  A little  Slightly  Somewhat  Very likely | | 0  1  2  3  4 | |  | |
|  | 9.3 | | How much do you feel confident that you are able to refuse it with a person they had known for 3 months? | | Not at all  A little  Slightly  Somewhat  Very likely | | 0  1  2  3  4 | |  | |
|  | 9.4 | | **How much do you feel** confident that you are able to refuse it with a person who offered them gift? | | Not at all  A little  Slightly  Somewhat  Very likely | | 0  1  2  3  4 | |  | |
|  | 9.5 | | **How much do you feel** confident that you are able to refuse to have sex with a person whom they care about deeply? | | Not at all  A little  Slightly  Somewhat  Very likely | | 0  1  2  3  4 | |  | |
|  | 9.6 | | **-How much do you feel** confident that you are able to refuse it with a person who paid for their school and training? | | Not at all  A little  Slightly  Somewhat  Very likely | | 0  1  2  3  4 | |  | |
|  | 9.7 | | -How much do you feel confident that you are able to refuse it with a person who had power over them? | | Not at all  A little  Slightly  Somewhat  Very likely | | 0  1  2  3  4 | |  | |
|  | 9.8 | | -How much do you feel confident that you could choose whom to have sex with? | | Not at all  A little  Slightly  Somewhat  Very likely | | 0  1  2  3  4 | |  | |
|  | 9.9 | | -How much do you feel **c**onfident that you could avoid sex if they wanted to? | | Not at all  A little  Slightly  Somewhat  Very likely | | 0  1  2  3  4 | |  | |
|  | **Self-efficacy in condom use** | | | | | | | | | |
|  | 9.10 | | -How much do you feel confident that could use condom correctly? | | Not at all  A little  Slightly  Somewhat  Very likely | | 0  1  2  3  4 | |  | |
|  | 9.11 | | -How much do you feel confident that could use condom every time that you have sex? | | Not at all  A little  Slightly  Somewhat  Very likely | | 0  1  2  3  4 | |  | |
|  | 9.13 | | How much do you feel confident that could use condom after you have been drinking? | | Not at all  A little  Slightly  Somewhat  Very likely | | 0  1  2  3  4 | |  | |
|  | 9.12 | | - How much do you feel confident that could insist on using a condom with a reluctant partner | | Not at all  A little  Slightly  Somewhat  Very likely | | 0  1  2  3  4 | |  | |
|  | 9.13 | | - How much did you feel confident that you could refuse sex if the partner does not want to use condom | | Not at all  A little  Slightly  Somewhat  Very likely | | 0  1  2  3  4 | |  | |
|  | ***Self0efficacy in communication with partner*** | | | | | | | | | |
|  |  | | Questions | | Category answers | | Code | | Skip | |
|  | 9.14 | | - How much do you feel confidence in persuading your previous/current partner to use condom? | | Not at all  A little  Slightly  Somewhat  Very likely | | 0  1  2  3  4 | |  | |
|  | 9.15 | | - How much do you feel confidence in asking your partner about other sex partners? | | Not at all  A little  Slightly  Somewhat  Very likely | | 0  1  2  3  4 | |  | |

**X. Peer sexual norms**

| **No** | **Peer sexual norms** | **Category answers** | **Code** | **Skip** |
| --- | --- | --- | --- | --- |
| 10.1 | What do you think how many of your friends having sexual intercourse? | None of my friends  Few of my friends  Some of my friends  Almost of my friends  All of my friends |  |  |
| 10.2 | What do you think how many of your friends having multiple sexual? | None of my friends  Few of my friends  Some of my friends  Almost of my friends  All of my friends |  |  |
| 10.3 | What do you think how many of your friends did not use condom? | None of my friends  Few of my friends  Some of my friends  Almost of my friends  All of my friends |  |  |
| 10.4 | What do you think how many of your friends selling sex? | None of my friends  Few of my friends  Some of my friends  Almost of my friends  All of my friends |  |  |
| 10.5 | What do you think how many of your friends having STIs? | None of my friends  Few of my friends  Some of my friends  Almost of my friends  All of my friends |  |  |
| 10.6 | What do you think how many of your friends using illicit drugs? | None of my friends  Few of my friends  Some of my friends  Almost of my friends  All of my friends |  |  |

**XI.** Exposure to STIs

|  | **A. Condition** | **B. Have you ever had?** | **C. in last 6 months?** | **D. do you currently have?** |
| --- | --- | --- | --- | --- |
| 11.1 | Ulcer or sore in penis/genital area | Yes 1  No 0→10.2 | Yes 1  No 0→10.2 | Yes 1  No 0 |
|  |  |  |  |  |
| 11.2 | Painful/burning sensation when urinating and intercourse | Yes 1  No 0→10.3 | Yes 1  No 0→4.15 | Yes 1  No 0 |
|  |  |  |  |  |
| 11.3 | Itching around the opening of the penis/vagina | Yes 1  No 0→4.17 | Yes 1  No 0→4.15 | Yes 1  No 0 |
|  |  |  |  |  |
| 11.4 | MAN: Genital discharge (a white, yellow, or green discharge from the penis)  WOMAN: increased vaginal discharge, white, yellow, or green color, with/without strong or foul odor. | Yes 1  No 0→Next Session | Yes 1  No 0→4.15 | Yes 1  No 0 |
| 11.5 | Warts in the vulva, vagina, anus | Yes 1  No 0→Next Session | Yes 1  No 0→4.15 | Yes 1  No 0 |

|  | Questions | Categories | Code | Skip |
| --- | --- | --- | --- | --- |
| 11.6 | Did you seek care from any health care provider/health facility because of these symptom(s) during the last time? | Yes  No | 1  0 |  |
| 11.7 | If not, what is (are) reason (s)? *(can choose many)* | It is normal  It is shameful  Mild, unworthy of medical attention  Mistrust of health facilities  Negative attitudes of health staff  Living far away the health facilities.  Lack of money  Others *(Please specify*)………… | 1  2  3  4  5  6  7  8 |  |
| 11.8 | Where did you go during the last time? | Private clinic  Pharmacy/drug store  Commune Health Station  District hospital  Provincial hospital  Central hospital  Traditional healer  Other *(Please specify*)…………….. | 1  2  3  4  5  6  7  8 |  |
| 11.9 | Why did you choose that place as the first one for treatment? (*can choose many*) | Confidentiality,  Convenient  Skilled provider Friendly provider  Cheap/Free of charge Recommended by friends/relatives  Good equipment Service available as desired  Prefer female examiner Other | 1  2  3  4  5  6  7  8  9  10 |  |

| **XII. Perception of risk to STIs** | | | | |
| --- | --- | --- | --- | --- |
|  | Questions | Categories | Code | Skip |
| 12.1 | -Do you think that you have the chance to get STIs? | no chance  small chance  somewhat chance  High" chance  Very high chance | 1  2  3  4  5 |  |
| 12.2 | -How much are you afraid of getting STIs? | no chance  small chance  somewhat chance  High" chance  Very high chance | 1  2  3  4  5 |  |
| 12.3 | --In your opinion, do you have a chance to get STIs in the future? | no chance  small chance  somewhat chance  High" chance  Very high chance | 1  2  3  4  5 |  |
| **XIII. Perception of risk to HIV/AIDS** | | | | |
|  | Questions | Categories | Code | Skip |
| 13.1 | -Do you think that you have the chance to get HIV/AIDS? | no chance  small chance  somewhat chance  High" chance  Very high chance | 1  2  3  4  5 |  |
| 13.2 | -How much are you afraid of getting HIV/AIDS? | no chance  small chance  somewhat chance  High" chance  Very high chance | 1  2  3  4  5 |  |
| 13.3 | --In your opinion, do you have a chance to get HIV/AIDS in the future? | no chance  small chance  somewhat chance  High" chance  Very high chance | 1  2  3  4  5 |  |

Thank you for your participation.
